# Disagreement between PCR and serological diagnosis of *Trypanosoma cruzi* infection in blood donors from a Colombian endemic region

**DOI:** 10.7705/biomedica.5441

**Published:** 2021-05-31

**Authors:** Liliana Torcoroma-García, Jhancy Rocío Aguilar, Marly Yojhana Bueno, Erika Marcela Moreno, Herminia Ramírez, Nelson Daza

**Affiliations:** 1 Programa de Maestría en Investigación en Enfermedades Infecciosas, Universidad de Santander, Bucaramanga, Colombia Universidad de Santander Programa de Maestría en Investigación en Enfermedades Infecciosas Universidad de Santander Bucaramanga Colombia; 2 Programa de Bacteriología y Laboratorio Clínico, Universidad de Santander, Bucaramanga, Colombia Universidad de Santander Programa de Bacteriología y Laboratorio Clínico Universidad de Santander Bucaramanga Colombia; 3 Escuela de Medicina, Universidad Industrial de Santander, Bucaramanga, Colombia Universidad Industrial de Santander Escuela de Medicina Universidad Industrial de Santander Bucaramanga Colombia

**Keywords:** Trypanosoma cruzi, Chagas disease, blood donors, serology, polymerase chain reaction, Trypanosoma cruzi, enfermedad de Chagas, donantes de sangre, serología, reacción en cadena de la polimerasa

## Abstract

**Introduction:**

Chagas' disease is the leading cause of infectious myocarditis worldwide. This infection caused by *Trypanosoma cruzi* is usually life-long and asymptomatic; however, the third part of infected people can develop severe or even fatal cardiomyopathy. As the parasitemia in the chronic phase is both low-grade and intermittent, *T. cruzi* infection is principally detected by serology, although this method has sensitivity and specificity limitations.

**Objective:**

To determine the level of agreement between serologic and molecular tests in 658 voluntary blood donors from six provinces in the Colombian department of Santander.

**Materials and methods:**

We evaluated an array of diagnostic technologies by cross-section sampling performing a serological double diagnostic test for *T. cruzi* antibody detection *(Chagas III ELISA™, BiosChile Group, and* ARCHITECT Chagas CMIA™, Abbott;, and DNA detection by polymerase chain reaction (PCR). We collected the demographic, clinical, and epidemiological information of participants. The sample size was calculated using Epidat™ and the statistical analysis was done with Stata 12.1™.

**Results:**

PCR was six times more sensitive in detecting *T. cruzi* infection than ELISA/CMIA with prevalence values of 1.8% (12/658) and 0.3% (2/658), respectively, and kappa=0.28 (95%CI: -0.03 - 0.59). In contrast, serology showed a sensitivity of 16.7% (95%CI: 2.09 -48.4) and a specificity of 100% (95%CI: 99.4 - 100). All seropositive samples were found to be positive by PCR.

**Conclusions:**

The implementation of PCR as a complementary method for screening donors could reduce the probability of false negative and the consequent risk of transfusional-transmission of Chagas' disease, especially in endemic regions.

## Resumen

**Introducción.:**

La enfermedad de Chagas constituye la principal causa de miocarditis infecciosa en el mundo. Causada por *Trypanosoma cruzi,* la infección puede persistir toda la vida de manera asintomática y silenciosa, pero un tercio de los infectados desarrolla cardiomiopatía grave. Debido a que la parasitemia en la fase crónica es baja e intermitente, el diagnóstico se hace principalmente mediante la detección de anticuerpos (serología), método que tiene limitaciones de sensibilidad y especificidad.

**Objetivo.:**

Determinar la concordancia entre el diagnóstico serológico y molecular de *T. cruzi* en 658 donantes voluntarios de sangre del departamento de Santander, Colombia.

**Materiales y métodos.:**

Se hizo un estudio de evaluación de tecnologías diagnósticas con muestreo transversal, utilizando un doble diagnóstico serológico para la detección de anticuerpos anti-T *cruzi (Chagas III ELISA™, BiosChile Group, y* ARCHITECT Chagas CMIA™, Abbott; y la de ADN por PCR. Se recolectó la información demográfica, clínica y epidemiológica de los participantes. El tamaño de la muestra se estimó utilizando Epidat™ y el análisis estadístico se hizo mediante Stata 12.1™.

**Resultados.:**

La sensibilidad de la PCR fue seis veces mayor que la de las pruebas de ELISA/CMIA, con prevalencias de 1,8 % (12/658) y 0,3 % (2/658), respectivamente, y kappa de 0,28 (IC_95%_ -0,03 - 0,59). La sensibilidad serológica fue de 16,7 % (IC_95%_ 2,09 - 48,4) y la especificidad de 100 % (IC_95%_ 99,4 - 100). Todas las muestras seropositivas fueron positivas también en la PCR.

**Conclusiones.:**

El uso de la PCR como método complementario para la tamización de donantes podría reducir el riesgo de falsos negativos y disminuir los casos de transmisión transfusional de la enfermedad de Chagas, especialmente en regiones endémicas.

Chagas' disease is an infection caused by the hemoflagellate protozoan *Trypanosoma cruzi* and it is considered one of the most significant neglected tropical diseases affecting 6 to 7 million individuals worldwide ^1^. In Latin America, this infection is reported as endemic in 21 countries leading to approximately 12,500 deaths per year and economic losses estimated at around USD$ 1.2 billion (almost five times greater than the regional malaria burden) ^2^^,^^3^. In the last few decades, this disease has become a global phenomenon, principally led by large-scale human migrations from endemic and rural regions to urban and non-endemic countries (Europe, USA, Canada, and Japan, among others) ^4^. In Colombia, the prevalence of *T. cruzi* infection has been estimated at 700,000 to 1,200,000 cases with the country's eastern departments being the most affected: Arauca (21.1%), Casanare (10%), and Santander (6.3%) ^5^.

*Trypanosoma cruzi* infection commonly exhibits a prolonged asymptomatic course (indeterminate phase), which can progress to severe chronic heart and digestive system disease or death in approximately 30% of cases. These clinical characteristics can delay the etiological diagnosis making it difficult to determine the real epidemiological impact of the infection.

Current Chagas' disease treatments are mainly aimed at the control of acute infection with good results in terms of parasite clearance. However, for the chronic phase, there is still no treatment to cure the infection or to prevent its progression ^6^. In the long term, the treatment in the indeterminate disease is most cost-effective than the therapy in the chronic phase when moderate to severe cardiomyopathy is established ^7^. In these cases, benznidazole decreases parasitic load and reduces heart injury ^7^. Consequently, it is important to rethink the epidemiological control measures for Chagas' disease and to develop and implement better methods for the detection of the etiological agent improving, therefore, the timely diagnosis and treatment of infected people before tissue damage or parasite transmission occurs ^8^.

As parasitemia is both low-grade and intermittent in the chronic phase of the infection, the diagnosis is based on the detection of anti-T *cruzi* by serological methods ^9^, but currently, there is not an ideal serological test or gold standard ^10^. However, the systematic screening of asymptomatic individuals (such as blood donors) is performed via the indirect detection of specific antibodies generated against the parasite in serum samples processed by methods such as ELISA and CMIA. Nevertheless, sensitivity and specificity limitations have been reported for this method in endemic regions where seronegativity has been described in patients diagnosed by direct methods and clinical findings (serosilent patients) ^11^^-^^14^. These disagreements between direct and serological techniques represent a serious risk of contagion, especially among recipients of blood or its components.

Molecular detection of the parasite's nuclear or kinetoplast DNA by PCR has been described as highly sensitive and specific and it is especially useful in the indeterminate and chronic phases of the infection. Unfortunately, the optimization and standardization of these methods have been difficult, with disagreements in results commonly found in comparison studies ^15^^-^^17^, and there is no commercial PCR assay for Chagas' disease available yet.

In comparative studies in blood donors, discordant results in serological and conventional, or even real-time PCR tests, have been reported in the third part of infected individuals ^8^^,^^15^^,^^18^. In enzootic infections as Chagas' disease, the seroconversion phenomena can occur in endemic areas associated with evolution adaptation mechanisms for parasite tolerance through parasitemia control or the absence of a fully-developed immune response, which is not necessarily equivalent to parasite clearance ^8^^,^^19^^,^^20^. Consequently, the parasitological cure could be established based on negative results in specific molecular tests more than by negative seroconversion ^8^.

In this context, we conducted a comparative study of the results obtained with two commercial serological (ELISA and CMIA) and molecular (in-house PCR) tests performed in 658 voluntary blood donors from the department of Santander, Colombia, an endemic region for Chagas' disease. The results of both assays were then compared showing a better performance of the molecular method in terms of sensitivity.

## Materials and methods

### Materials

We used a QIAamp DNA Blood MiniKit™ (Qiagen) for whole DNA extraction from blood. GoTaq Flexi™, dNTPs Mix™, 5X Buffer GoTaq Flexi™, and 25 mM of MgCl were purchased from Promega. We also used a Mastercycler Nexus™ (Eppendorf), a Wide Mini-Sub Cell GT Cell Horizontal Electrophoresis Chamber™, and a NanoDrop ND-1000 UV-VIS Spectrophotometer™ (Thermo Fisher Scientific). The PowerPac HC™ and the Gel Doc XR +Gel Documentation System™ were acquired from Bio-Rad. The Gene Ruler 1 kb and the 6X DNA Loading Dye were purchased from Thermo Fisher Scientific. Agarose and SYBR Safe DNA Gel Stain™ were purchased from Invitrogen Life Technologies. All the reagents and the water used were ultrapure.

### Study design

We evaluated the diagnostic technologies by cross-sectional sampling performing a serological double diagnostic for *T. cruzi* antibody detection and DNA detection by PCR. Blood samples were prospectively collected from 658 voluntary donors who had participated in blood donation campaigns organized by a blood bank from a third-level hospital in Santander.

All the volunteers completed two self-reported questionnaires: one for blood bank-donor selection and the other to collect specific information for the study. To prevent unintentional double registrations, we excluded individuals previously sampled in the study. Relevant demographic, clinical, and epidemiological information was also collected. Consecutive sampling was performed over approximately twelve months (June, 2013, to May, 2014) until reaching the previously-determined sample size, which was calculated using Epidat 4.1™ software taking into consideration the reported prevalence for *T. cruzi* infection in the blood bank (5%) with 95% confidence level and 5% minimum precision.

### Inclusion and exclusion criteria

We selected individuals who signed the informed consent, complied with the Colombian blood donor standards, and provided all the specific information required for the study variables.

### Blood samples

Whole blood and serum samples for molecular and serological tests were collected on the same day by phlebotomy in 5 ml Vacutainer™ sterile tubes with EDTA or without anticoagulant, respectively. Serum samples were separated within two hours of collection and kept at -20°C until serological processing. EDTA-supplemented blood samples were preserved at 4°C and protected from light until DNA extraction. The serology and DNA extraction procedures were completed within 15 days of collection.

### DNA extraction

DNA extraction from EDTA-supplemented blood samples was performed using a QIAmp DNA Blood Mini Kit™ (QIAGEN) following the manufacturers' protocol slightly modified. Briefly, 200 μl of blood were washed twice with STE solution (100 mM NaCl; 10 mM Tris-Cl, pH 8.0; 1 mM EDTA) on the sample-equilibrated column to reduce the ionic strength and to optimize DNA extraction. The quality of the extracted DNA was confirmed using 1% agarose electrophoresis and its concentration was determined on a NanoDrop ND-1000 UV-VIS Spectrophotometer™ (Thermo Fisher Scientific), and quantified with the NanoDrop 2000/2000c Spectrophotometer Software™ (Thermo Fisher Scientific). As controls, we used 300 μl of each whole blood sample on Whatman No. 3 filter paper stored in 1.5 ml sterile plastic tubes at -20°C.

### Polymerase chain reaction (PCR)

PCR assays were performed using the forward TckDNA121F (5'-AAATAATGTACGGGGGAGATGCATGA-3) and the reverse TckDNA122R (5'-GGTTCGATTGGGGTTGGTGTAATATA-3') primer sequences described by Sturm, *et al.* (21). These oligonucleotides were designed based on conserved regions of the *T. cruzi* 9813 strain and V101 clone kinetoplast DNA (GenBank accession number 311875422) and synthesized by Invitrogen Life Technologies.

For the PCR, we used total DNA and control samples from patients as templates. We optimized the PCR reagent concentrations and running conditions based on amplicon efficiency and specificity parameters. The positive DNA controls used for the PCR assays were: a) genomic material extracted from an axenic LIT culture of *T. cruzi* I (TcI) SYLVIO-X10 strain epimastigotes, and b) total DNA isolated from EDTA-blood samples obtained from clinically confirmed patients with chagasic cardiomyopathy. All samples were processed in duplicate in two independent experiments and all positive PCR samples were confirmed in triplicate.

The quality, sensitivity, and specificity of the DNA amplification were assessed using 1.5% DNA agarose electrophoresis assays stained with 1X SYBR Safe™ (Invitrogen Life Technologies), then visualized using a 302 nm UV transilluminator, and documented with a Gel Doc XR+ Gel Documentation System™ (BioRad).

### Anti-T. cruzi antibodies

We screened the serum samples for anti*-T. cruzi* antibodies using a *Chagas III* ELISA Kit™ *(BiosChile Group) and* retested using an ARCHITECT Chagas CMIA Kit™ (Abbott) following the manufacturer's instructions. Samples with results above the cutoff value in both ELISA and CMIA tests were considered positive and confirmed by Western Blot. The absorbance cutoff values were below 0.3 in all the analyses performed. Sera from patients with chronic chagasic cardiomyopathy were utilized as positive controls.

### Statistical analysis

The personal, clinical, and epidemiological data of the donors, as well as their serological and PCR results, were recorded in a database with the Epidata 3.1. software. Frequency distribution, central tendency, and dispersion statistics were calculated for the quantitative variables and proportions for the qualitative variables. The association between the independent variables and the infection and the normality of the continuous data were evaluated using the chi-square and Shapiro Wilk tests, respectively. The agreement between ELISA/CMIA and PCR was calculated by means of the kappa coefficient. The sensitivity, specificity, positive predictive value (PPV), and negative predictive value (NPV) of the serological tests were calculated taking the PCR as the defined reference test. All statistical analyses were carried out using Stata 12.1™.

### Ethical considerations

The study was approved by the Ethics and Research Committee at *Universidad de Santander* and by the Ethics Committee of the hospital to which the blood bank is affiliated and conducted according to the current Colombian legislation (Resolution 8430 of 1993) and the Helsinki Declaration. All participants signed an informed consent form.

To guarantee confidential data handling, information was coded. All assays using donor samples were performed in accordance with defined quality control standards. Blood donors identified during screening as *T. cruzi* seropositive were referred for medical attention and follow-up testing including hemoculture and Western Blot.

## Results

### Study population

The main demographic characteristics of the study population (place of residence, sex, age, and occupation) are shown in table 1. The study population (658 voluntary donors) was composed mainly of young people (50% aged 18 to 24, average age: 28.9 years ± 10.1 SD), males (59.4%, n=391), O blood type (61.2%, n=403), Rh+ (60.5%, n=399), students (56.5%, n=372), and residents in several Santander municipalities (93.3%, n=614) 75% of them located in Chagas' disease-endemic areas.

**Table 1:** Demographic characteristics of the study population

Department	Province	Sex						Age	Occupation*					
		n	%	F		M		X SD	Student		Employed		Unemployed^1^	
				n	%	n	%		n	%	n	%	n	%
Santander		614	93.3	257	39.0	357	54.2	29.4 ± 10.3	359	54.5	233	35.4	10	1.5
	Comunera	164	24.9	75	11.3	89	13.5	26.0 ± 7.0	127	19.3	33	5.0	2	0.3
	García Rovira	8	1.2	-	-	8	1.2	24.2 ± 2.8	8	1.2	-	-	-	-
	Guanentá	380	57.7	168	25.5	212	32.2	31.7 ± 11.2	196	29.8	166	25.2	8	1.2
	Metropolitana	21	3.2	6	0.9	15	2.3	25.7 ± 6.7	9	1.4	12	1.8	-	-
	Soto Norte	1	0.2	1	0.2	-	-	22.0 -	1	0.2	-	-	-	-
	Vélez	1	0.2	-	-	1	0.2	21.0 -	-	-	1	0.2	-	-
	Yariguíes	28	4.3	4	0.6	24	3.6	22.0 ± 1.4	15	2.3	13	2.0	-	-
	Not reported	11	1.7	3	0.5	8	1.2	29.1 ± 13.8	3	0.5	8	1.2	-	-
Other departments^2^	-	27	4.1	6	0.9	21	3.2	22.7 ± 1.7	13	2.0	12	1.8	-	-
Missing data	-	17	2.6	4	0.6	13	2.0	--	-	-	-	-	-	-
		658	100	267	40.6	391	59.4	28.9 ± 10.1	372	56.5	245	37.2	10	1.5

n: Number; F: Female; M: Male

* 31 Missing data

^1^ Unemployed: Retired and housewife people

^2^ Other Colombian departments: Endemic [Boyacá 1.4% (n=9), Bolivar 1.1% (n=7), Magdalena 0.3% (n=2); Cesar 0.3% (n=2); Casanare 0.2% (n=1), Sucre 0.2% (n=1)], and non-endemics (n=5)

**Figure 1:**
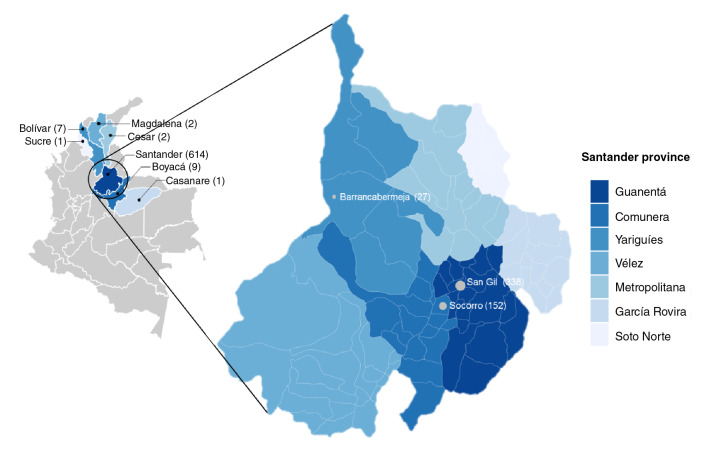
Colombian map of sample collection sites and zoom into Santander department and its provinces.

The provinces of Guanentá (n=380), Comunera (n=164), and Yariguíes (n=28) provided 86.9% (n=572) of the collected samples, mainly from the towns of San Gil (n=338), Socorro (n=152), and Barrancabermeja (n=27), correspondingly (figure 1). The remaining donors (3.3% non-residents) came from other Colombian departments also endemic for Chagas' disease according to the National Public Health Surveillance System (6): 1.4% (n=9) from Boyacá; 1.1% (n=7) from Bolívar; 0.3% (n=2) from Magdalena; 0.3% (n=2) from Cesar; 0.2% (n=1) from Casanare, and 0.2% (n=1) from Sucre. The residence data of 17 donors were missing.

Risk factors were only reported by 7.1% (n=47) of the participants (table 2). Only 40.7% (n=268) had donated blood previously and a very low percentage (0.6%, n=4) had been bitten by a triatomine bug at some point in their lives.

**Table 2:** Association between risk factors for transfusion-transmissible infections and PCR positive results for *Trypanosoma cruzi*

Variable	n=658	%	PCR (+)/(-)	p value*
Tattoo <12 months	18	2.8	0/18	0.56
Travel to endemic regions^1^ <6 months	8	1.2	0/8	0.70
Transfusion <12 months	5	0.8	0/5	0.76
Biological accident <6 months	9	1.4	0/9	0.68
Hallucinogenic drug use <12 months	5	0.8	0/5	0.76
Bitten by kissing bug (triatomine)	4	0.6	1/3	0.001

^1^ Endemic regions for leishmaniasis, dengue, malaria or yellow fever

* p<0.05: statistically significant

### DNA isolation and amplification

**Figure 2:**
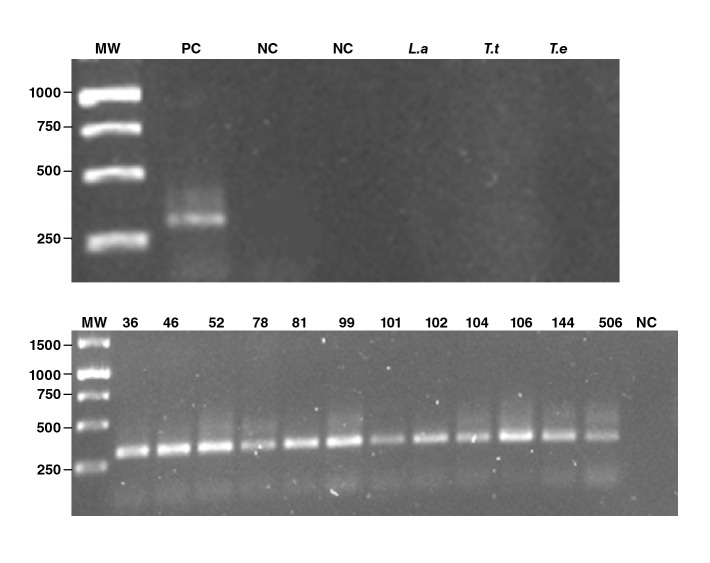
PCR products in 1.5% agarose electrophoresis gel. **A.** Control DNA samples. **B.** Positive PCR assays form donor DNA samples (36, 46, 52, 78, 81, 99, 101, 102, 104, 106, 144, and 506).

MW: Molecular Weight Gene Ruler 1Kb DNA Ladder; PC: Positive control *(Trypanosoma cruzi* culture total DNA extraction); NC: Negative control;: L.a: *Leishmania amazonesis.*T.t: *Trypanosoma teilheri;* T.e: *Trypanosoma evansi*

For PCR assays, total DNA was isolated from whole blood samples and concentrations between 40 and 70 ng/μl were obtained and used as templates. The amplification assays were performed using the oligonucleotide pair described by Sturm, *et al.*^21^. To increase the 330 bp *T. cruzi-*kDNA amplicon production and the specificity of the test, the reagent concentrations and cycling parameters used were optimized as indicated in the supplementary material (table S1). The sensitivity of the optimized PCR was estimated at 12.5ng/μl of DNA (equivalent to 1 cell/μl) using total DNA samples isolated from *T. cruzi* cultures as templates at various concentrations. Non-target-size bands were generated employing *T. theleri, T. evansi,* and *Leishmania amazonensis* genomic material as DNA templates to confirm a 100% specificity for the optimized test (figure 2A).

### Serological vs molecular test

We found 0.3% (n=2) of samples positive for *T. cruzi* antibodies in serum with both serological methods *(Chagas III* ELISA Kit™ from *BiosChile Group and* ARCHITECT Chagas CMIA Kit™ from Abbott). Conversely, in the molecular analysis, we found a total of 12 samples (1.8%) (figure 2B). We did not find false positives as all the positive serological results (n=2) also exhibited the specific amplicon band by PCR thus allowing us to estimate a 100% specificity for the serological techniques used. According to the PCR results, a 1.8% (n=12) *T. cruzi* infection prevalence was calculated for the study population. Using the kappa coefficient, the agreement between the serological and molecular results was calculated at 0.28 (95%CI: -0.03 - 0.59) and the following serology parameters were estimated using the molecular assay as the defined reference test: Sensitivity, 16.7% (95%CI: 2.09 - 48.4); specificity, 100% (95%CI: 99.4 - 100); PPV, 100% (95%CI: 15.8 - 100), and NPV, 98.5% (95%CI: 97.2 - 99.3) (table 3).

**Table 3:** Sensitivity, specificity, predictive values and concordance of serological tests as compared to PCR for the diagnosis of *Trypanosoma cruzi* infection in blood donors from a Colombian endemic region

Diagnostic test*	PCR (+)	PCR (-)	Total
Serological			
(+)	2	0	2
(-)	10	646	656
Total	12	646	658

*Kappa coefficient: 0.28 (95%IC: -0.03-0.59); sensitivity: 16.7% (95%CI: 2.09-48.4); specificity: 100% (95%CI: 99.4-100); positive predictive value (PPV) 100% (95%CI: 15.8-100), and negative predictive value (NPV) 98.5% (95%CI: 97.2-99.3)

To determine possible associations, the twelve donors who tested positive by either PCR or serology were characterized using parameters such as their place of residence, sex, occupation, and relevant epidemiologic backgrounds (triatomine bite or previous donation) (table 2). It is noteworthy that 83% (10/12) of the individuals who tested positive for *T. cruzi* infection by PCR came from the province of Guanenta (San Gil), 75% (9/12) were male, and 33% (4/12) had previously donated blood. One of the two individuals who tested positive by both serology and PCR assays (sample 78) reported having previously suffered a triatomine bite (table 2).

## Discussion

In endemic areas of many Latin American countries, *T. cruzi* is primarily transmitted by contact with the feces of infected blood-sucking triatomine bugs (vector-borne). However, transmission by transfusion remains significant, principally due to the dispersal of the infection beyond the geographical borders of insect vectors, which alters the infection's epidemiological dynamics ^4^. Control efforts in the region have been centered on vector control and the implementation of protocols and regulations for compulsory blood donor screening. The prevalence of *T. cruzi* infection among blood bank donors has shown a great variation, with rates varying between 1.3% and 51% ^22^. In Colombia, the seroprevalence in blood donors has been reported between 0.09% and 2.1% depending significantly on the region studied ^23^^-^^28^, with an estimated 5% in the nation's general population ^27^. In Santander, the *T. cruzi* infection rate for voluntary blood donors has been reported at around 0.53% ^28^.

Currently, blood safety depends primarily on the sensitivity of the laboratory screening tests, as well as on the implementation of progressively more stringent donor eligibility criteria. However, due to the largely asymptomatic course of *T. cruzi* infection and its characteristic low parasitemia, in its late stages, the diagnosis is traditionally carried out using two or more serologic tests for specific IgG antibody detection ^9^. In blood bank screening, the most commonly-used methods are those based on enzyme-linked immunosorbent assays (ELISA) and chemiluminescent microparticle immunoassays (CMIA), with more complex techniques, such as indirect immunofluorescence (IFI) and immunoblotting, generally being used for confirmatory purposes. Previously, high specificity and sensitivity levels (up to 95% of IFI standards) have been reported for ELISA and CMIA assays, as well as a high reproducibility of ELISA, IFI, and indirect hemagglutination (IHA) ^29^^,^^30^, but they also exhibit limitations that should be considered and analyzed through comparative studies with modern complementary techniques ^29^. For example, the false seropositive results reported in these tests have been explained by the cross-reactivity with other hemoflagelate protozoa such as *Leishmania* spp. or *Trypanosoma rangeli*^29^^,^^31^. Additionally, and most preoccupying, multiple studies have reported seronegativity in patients who are symptomatic for Chagas' disease and exhibit molecular evidence of *T. cruzi* infection (i. e. who are PCR-positive) ^12^^,^^31^^,^^32^.

Here we conducted a comparative study of two methods for serological screening of specific *T. cruzi* antibodies *(Chagas IIIELISA Kit™ from BiosChile Group and* ARCHITECT Chagas CMIA Kit™ from Abbott) and a molecular technique using the in-house PCR protocol previously described by Sturm, *et al.*^21^. The tests were applied to 658 voluntary blood donors from a Chagas' disease-endemic region (Santander, Colombia) with a reported 6.3% *T. cruzi* infection prevalence ^5^ and great diversity in domestic and wild triatomine insects (11 different species of which seven are involved in Chagas' disease transmission) ^5^^,^^33^.

Our results showed that the molecular method was six times more sensitive than the ELISA and CMIA assays at detecting *T. cruzi* infection in blood samples. These results are in agreement with previous studies documenting a better performance for molecular methods in terms of sensitivity and specificity, especially for parasite detection in blood samples from chronic patients and cases of doubtful serological tests ^8^^,^^11^^-^^14^^,^^18^^,^^31^. Thus, in patients with cardiac ^12^ and/or digestive abnormalities ^13^, which are characteristic of chagasic infection with low parasitemia, PCR appeared to be an efficient method for *T. cruzi* DNA detection even for doubtful ^8^^,^^11^ or serologically negative cases ^8^^,^^12^^,^^13^.

Comparative studies of serological results and molecular PCR test performed in a cross-sectional blood donor cohort evidenced low antibody levels or discordant test results in one third of the seropositive samples ^8^^,^^18^. These results were explained by parasite tissue sequestration after the resolution of acute infection leading to a low-grade and intermittent parasitemia in indeterminate and chronic infection stages ^8^^,^^18^^-^^20^. These phenomena can be also accompanied by a low antigenic stimulus with a resulting low antibody production or even seroconversion ^19^^-^^20^. The association of antibody levels with peripheral parasitemia was previously described in blood donor studies ^8^^,^^15^, parameters that were also correlated with the severity of the chagasic cardiomyopathy ^8^. These authors concluded that in endemic areas a minor proportion of the infected individuals are able to control the circulating parasite in the blood ^8^, but does not necessarily represent its elimination. Consequently, the absence of reliable biomarkers to identify the parasite persistence is a major obstacle to understand the natural history of chagasic cardiomyopathy and determine the true impact of the disease.

The performance of the molecular method can be explained by the fact that PCR is based on direct detection of parasite DNA while immunological tests are strongly dependent on the patient's immune status and the time since infection. They also rely heavily on the technical conditions under which the assays are performed (for example, antigen, parasite lineage, or developmental stage, among others) ^31^^,^^32^. Therefore, despite chronic infection, some patients are unable to develop an antibody response detectable by conventional serology. This phenomenon could be more common than expected as revealed by previous reports in endemic nations such as Argentina, Bolivia, Venezuela, and Brazil ^12^^,^^13^^,^^33^. Some of its possible causes include: a) window-phase or very low antibody titers in the early stage of infection ^34^; b) inability of some *T. cruzi* strains to induce specific antibody production ^35^; c) HIV co-infection causing decreased antigen presentation due to virus tropism on CD4+ T-, dendritic, and natural killer cells, and the consequent impairment of their antibody secretion ^36^; d) non-responder phenomenon or persistent antibody-negative (immunosilent) carriers who are unable to produce a detectable humoral response and present appropriate antigens (possibly associated with CD4+ and CD8+ T-cell depletion due to the affinity of the thymus as a *T.* cruzi-infection target), as well as the reduction of IL-2 (a pivotal cytokine in cellular memory immunity and antigen recognition) ^34^^,^^35^, and e) procedural testing errors.

Currently, PCR protocols using different targets such as repeated satellite sequences ^11^, Tc24 flagella proteins 3'end ORF ^37^, or kinetoplast DNA ^21^ have been described. The PCR described by Sturm, *et al.*^21^, uses a specific primer pair for the amplification of a 330 bp fragment from kDNA minicircles resulting in a highly sensitive (70% to 100%) and specific (96.5% to 100%) method due to the high copy-number of the mitochondrial genome (10,000 to 30,000 minicircles and their respective four copies in the variable region). Additionally, this protocol was also reported to exhibit specificity of 100% compared to other kinetoplastid genera (*Crithidia luciliae, Leptomonas collosoma, Herpetomonas mariadeanei, Endotrypanum* spp., *Blastocrithidia culicis, Leishmania tarentolae,* and *T. rangeli)*^21^, which explains why we selected and optimized the method to use it as the molecular test. The modified PCR results in an increase in sensitivity and specificity rates.

In the present study, all positive samples by serology (2/658) were also positive by PCR (two true positive results), however, 10 samples were only found positive using the molecular assay (10 false negatives by serology). Given that four of the PCR-positive donors had previously donated blood, an apparent epidemiological risk is evident for transfusion-transmission associated with possible false negatives from serology.

In the correlation analysis of *T. cruzi* PCR-positive donors and the independent variables (table 2), the triatomine bite was the only variable that showed a statistically significant association (p<0.001); this risk factor was reported by 33.3% (4/12) of the PCR-positive individuals and 10/12 (83.3%) of these positive donors were residents in Guanentá, a highly endemic area for Chagas' disease (table 1).

Regarding serological methods, 96% sensitivity and 99% specificity levels have been estimated for screening ELISA assays ^38^. Specifically for the Chagas III ELISA kit from the BiosChile Group an 82% to 99% specificity and a 99% sensitivity have been reported ^39^^,^^40^, and the test has also shown an excellent concordance with other ELISA tests, such as the Chagas test IIC V.1 (Research Institute in Health Sciences at *Universidad Nacional de Asunción,* Paraguay) and the Chagatest ELISA (Wiener) ^40^, with a kappa index of 0.92. However, in aboriginal communities in the Venezuelan States of Bolívar and Delta Amacuro, the ELISA Chagas III (BiosChile) method had a poor performance in detecting specific *T. cruzi-IgG* antibodies ^41^. A significant performance disagreement has also been reported for commercial ELISA results for *T. cruzi* antibody detection. In this regard, Guzmán-Gómez, et al. ^42^, analyzed 37 samples using five serological techniques and found that only one sample was positive with all techniques: one with four and nine with three. All samples were positive for at least two of the tests used but in a matrix of various combinations ^42^.

It is important to acknowledge that the population recruited in blood donor campaigns (as in this study) are demographically different from the conventional donors routinely attending blood banks and both of them differ from the general population. Therefore, the prevalence registered in our study cannot be considered the Chagas' disease prevalence in the general population, mainly because the volunteer participants were mostly males, students, young, and unemployed (table 1); besides they were in good health and had healthy personal habits ^28^. It is possible, then, that a higher prevalence would be found in the general population in Santander from all age and sex groups, as well as normal occupational diversity.

However, our purposes were first, to compare the diagnostic utility of ELISA/CMIA and PCR techniques for Chagas' disease diagnosis; second, to determine the existence of a possible serosilent phenomenon in blood donors from Santander municipalities, and third, to emphasize the need to rethink progressively more restrictive donor eligibility criteria and increasingly sensitive blood donor screening methods like PCR. The implementation of a PCR test, as described herein, could be used as a complementary method for screening blood donors thus reducing the risk of false-negative results and Chagas' disease transmission by transfusion, especially in endemic regions such as the department of Santander in Colombia.

## Supplementary files

**Table S1:** PCR parameters for *Trypanosoma cruzi* kDNA amplification

Reagent parameters		
Reagent	This study	Sturm, et al., 1989
MgCl	2.5 mM	-
KCl	50 mM	50 mM
Tris-HCl (pH 8.8)	10 mM	10 mM
dNTP mix	0.8 mM	1.5mM
Dithiothreitol	-	1mM
Sense primer	0.4 μM	NR
Antisense primer	0.4 μM	NR
*Taq* DNA polymerase	0.1 U/μl	0.02-0.08 U/μl
DNA template	100 nM	NR
Cycling parameters		
Step	Parameter	Parameter
Initial denaturalization	5 minutes at 94°C	2 minutes at 94°C
Cycles	34X	34X
Denaturalization	40 seconds at 94°C	1 minute at 93°C;
Hybridization	40 seconds at 55°C	1 minutes at 37°C
Elongation	50 seconds at 72°C	1-2 minutes at 70°C
Final extension	10 minutes at 72°C	1-2 minutes at 70°C

NR: Not reported
